# The role of intestinal macrophage polarization in colitis-associated colon cancer

**DOI:** 10.3389/fimmu.2025.1537631

**Published:** 2025-03-05

**Authors:** Yujie Deng, Xiaobing Jia, Liu Liu, Qiao He, Lei Liu

**Affiliations:** ^1^ Medical Research Center, The Third People’s Hospital of Chengdu (Affiliated Hospital of Southwest Jiaotong University), College of Medicine, Southwest Jiaotong University, Chengdu, Sichuan, China; ^2^ The First Outpatient Department, The General Hospital of Western Theater Command, Chengdu, Sichuan, China; ^3^ Department of Gastroenterology, Affiliated Hospital of Southwest Jiaotong University, The Third People’s Hospital of Chengdu, Chengdu, Sichuan, China; ^4^ Department of Clinical Laboratory, Sichuan Cancer Hospital & Institute, Sichuan Cancer Center, Affiliated Cancer Hospital of University of Electronic Scie Technology of China, Chengdu, Sichuan, China; ^5^ Medical Research Center, Affiliated Hospital of Southwest Jiaotong University, The Third People’s Hospital of Chengdu, Chengdu, Sichuan, China

**Keywords:** macrophage polarization, inflammatory bowel disease, colitis-associated colon cancer, immunotherapy, tumor-associated macrophages (TAMs)

## Abstract

Chronic inflammation of the intestine is a significant risk factor in the development of colorectal cancer. The emergence of colitis and colorectal cancer is a complex, multifactorial process involving chronic inflammation, immune regulation, and tumor microenvironment remodeling. Macrophages represent one of the most prevalent cells in the colorectal cancer microenvironment and play a pivotal role in maintaining intestinal health and the development of colitis-associated colon cancer (CAC). Macrophages are activated mainly in two ways and resulted in three phenotypes: classically activated macrophages (M1), alternatively activated macrophages (M2). The most characteristic of these cells are the pro-inflammatory M1 and anti-inflammatory M2 types, which play different roles at different stages of the disease. During chronic inflammation progresses to cancer, the proportion of M2 macrophages gradually increases. The M2 macrophages secrete cytokines such as IL-10 and TGF-β, which promote angiogenesis and matrix remodeling, and create the favorable conditions for cancer cell proliferation, infiltration, and migration. Therefore, macrophage polarization has a dual effect on the progression of colitis to CAC. The combination of immunotherapy with reprogrammed macrophages and anti-tumor drugs may provide an effective means for enhancing the therapeutic effect. It may represent a promising avenue for developing novel treatments for CAC. In this review, we focus on the process of intestinal macrophage polarization in CAC and the role of intestinal macrophage polarization in the progression of colitis to colon cancer, and review the immunotherapy targets and relevant drugs targeting macrophages in CAC.

## 1 Introduction

Colorectal cancer (CRC) can bev defined as one of the most common malignant tumors of the digestive tract. According to data from the Global Cancer Observatory (GLOBOCAN) 2022, CRC ranks the third in incidence and the second in mortality among all malignancies worldwide (1). Chronic bowel inflammation is known to be a major risk factor for developing to CRC. As the largest digestive organ in the human body, the intestines have a highly developed immune system, which are relatively tolerant and allowing the intestines to adapt to constant exposure to foodborne pathogens. Therefore, intestinal immunity is critical in protecting the intestinal barrier and preventing intestinal diseases. Macrophages represent a crucial component of the immune system in the intestine. These cells form a dense network along the digestive tract and perform a pivotal function in maintaining the equilibrium of the microbial population on the intestinal mucosal surface and facilitating the continuous renewal of intestinal epithelial cells. As the first line of leukocyte defense, intestinal macrophages protect against pathogens that invade the inner layers of the intestine. They maintain tissue homeostasis by secreting bioactive substances and regulating immune responses (2). Macrophages also participate in the pathological processes of inflammatory bowel disease (IBD) and CRC. An increasing number of studies have demonstrated that tumor-associated macrophages (TAMs) disrupt the homeostasis of the intestinal environment and are significantly associated with tumor invasion, infiltration, and metastasis (3). In addition, the currently used animal models for CRC study are mainly the CAC models, such as AOM/DSS induced CAC, so it is essential to understand the role of intestinal macrophages in the progress of colitis to CAC. In this review, we discuss the role of intestinal macrophages in the pathogenesis of colitis-associated colorectal cancer, focusing on the differential effects and imbalance of M1 and M2 macrophages on the immune pathophysiology of CAC. It also highlights the metabolites, cytokines, and microbiota involved in inducing the polarization of TAMs and prospects for therapeutic drugs targeting macrophages.

## 2 Macrophages in the intestine

### 2.1 Origin of intestinal macrophages

The intestine contains the most abundant reservoir of macrophages. In humans, tissue macrophages arise from hematopoietic and embryonic precursors. In contrast to most tissues, intestinal macrophages are derived primarily from innate macrophages that exist before birth and continuously replenished by circulating monocytes in adulthood (4). These cells coexist and collaborate in intestinal tissue (5). Human intestinal macrophages derive CD11c^+^CD14^hi^ monocytes from the bloodstream, which undergo a series of differentiation processes upon entering the intestine to become mature macrophages with low expression of CD11c and CD14 and high expression of MHCII, CD206, and CD163 (6, 7). However, it is unclear whether a small proportion of embryonic-derived self-maintaining macrophages exist in the adult gut. Like humans, monocytes in the intestines of mice proliferate during the embryonic to neonatal period. After weaning, however, homeostatic intestinal macrophages in mice are primarily replenished by circulating monocytes characterized by CC-chemokine receptor 2-high (CCR2^hi^), lymphocyte antigen 6C-high (LY6C^hi^), MHCII^-^, CX3C^-^chemokine receptor 1-low (CX3CR1^low^) monocytes. When these circulating monocytes enter the intestinal lamina propria, they acquire MHCII and lose Ly6C expression. Following this, they upregulate F4/80, CD64, and CX3CR1 and differentiated into mature Ly6C^-^MHCII^hi^CX3CR1^hi^ macrophages (7, 8). In addition, A CX3CR1^hi^ CD4^+^TIM4^+^ macrophage subset has been identified in submucosa and external muscle layer in mice, which exhibits specific surface markers. These cells are demonstrated to have the capacity for self-renewal and independent maintenance of the local macrophage population through monocyte recruitment (9).

### 2.2 The homeostasis of intestinal macrophages

**Figure 1:**
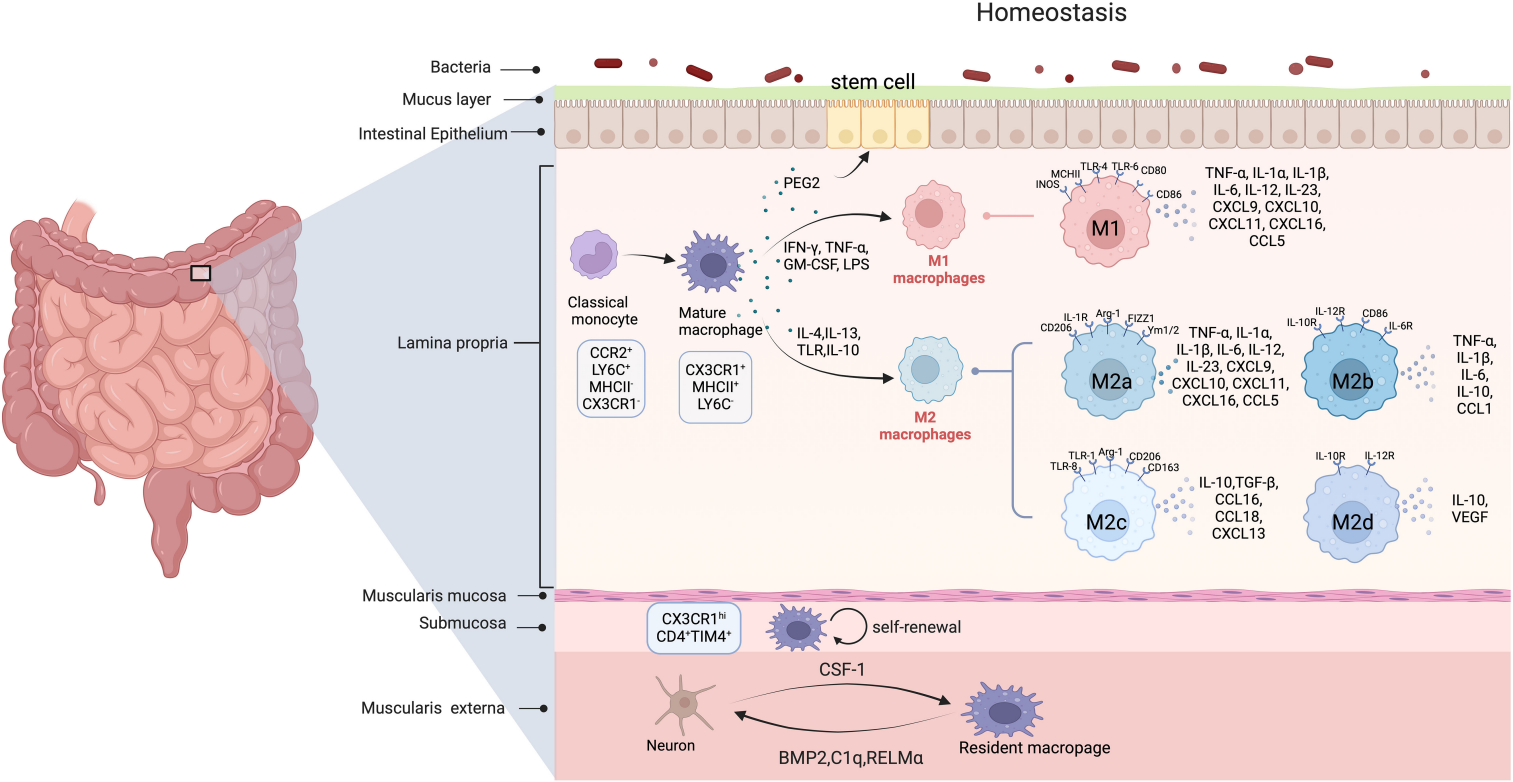
The origin and polarization of intestinal macrophages in homeostasis. *(Created in BioRender. yujie, D* (2025). *https://BioRender.com/r15b808* *)* In homeostasis, intestinal macrophages are largely replenished by circulating CCR2^hi^, LY6C^hi^, MHCII^–^, and CX3CR1^low^monocytes. Submucosa and muscularis externa in mice contain self-renewing macrophages as well. Muscularis externa macrophages affect intestinal peristalsis by secreting BMP2, RELM-α, and C1q, and aid enteric neuron differentiation, while enteric neurons release CSF1 to sustain nearby macrophages. Mature macrophages polarize into the pro-inflammatory M1 phenotype upon stimulation by Th1 cytokines (e.g., IFN-γ) and TLR ligands such as LPS. These M1 macrophages secrete high levels of pro-inflammatory cytokines (e.g., TNF-α, IL-1β, and IL-6), which initiate and sustain inflammatory responses to eliminate pathogens in acute enteritis. In contrast, exposure to Th2 cytokines (e.g., IL-4 and IL-13) drives macrophage polarization into distinct M2 subtypes: M2a macrophages primarily mediate tissue repair, immunosuppression, and allergic reactions; M2b macrophages regulate immune homeostasis; while M2c and M2d subtypes exhibit potent immunosuppressive functions through mechanisms such as apoptotic cell clearance and cytokine-mediated T cell inhibition. (In this figure, curved arrows indicate secretion or promotion and circular arrows represent self-renewal.).

Intestinal macrophages regulate the homeostasis of the gut environment by secreting various bioactive substances (**Figure 1**). In a steady state, macrophages inherently exhibit low levels of TNF-alpha, which plays a regulatory role in the proliferation of intestinal epithelial cells, the maintenance of the intestinal epithelial barrier, and the production of tissue remodeling proteins in intestinal mesenchymal cells (10). These intestinal macrophages display a M2-like phenotype, characterized by the production of anti-inflammatory molecules such as IL-10 and TGF-β, reduction expression of pro-inflammatory mediators like IL-6 and iNOS, diminishing responsiveness to Toll-like receptor (TLR) stimulation, and facilitation of regulatory T cell (Treg) expansion (11). In addition, intestinal macrophages interact closely with intestinal epithelial cells. The macrophages promote the renewal and integrity of intestinal epithelial cells by producing factors such as prostaglandin E2 (PGE2) and hepatocyte growth factor (HGF) (12). With the microbial-drove stimulation, macrophages produce IL-1β, which promotes type 3 innate lymphoid cells (ILC3) to release CSF2 and stimulate macrophages to secrete IL-10. Previous studies have shown that macrophage-derived IL-10 is crucial for maintaining and expanding antigen-specific Treg cells in the intestinal mucosa of mice, which helps to stabilize immune responses in the gut (13, 14). Furthermore, macrophages in the mucosa and submucosa also form a tight bond with the endothelial cells, thereby supporting their maintenance by producing vascular endothelial growth factors (e.g., VEGF-C) (15).

### 2.3 Factors influencing intestinal macrophages development

The function of macrophages is influenced by various factors, including multiple intestinal cells, the microbiota, and neuro-immune interactions that regulate intestinal cellular activity (16). Intestinal epithelial cells are situated at the interface between intestinal symbionts and macrophages (17). It comprises different types of specialized epithelial cells, such as enterocytes, Paneth cells, goblet cells, endocytes, and microfold cells (18). In intestinal epithelial cells, the mucus layer overlays the intestinal mucosa, the glycocalyx present on the microvilli of absorptive epithelial cells, and the tight junctions linking these cells collectively constitute a physical barrier. This barrier protects the intestinal mucosa from the gut microbiota and invading pathogens (19). In addition to isolating the gut microbiota from host immune cells, intestinal epithelial cells are stimulated by the gut microbiota and derived factors, such as IL-18 and chemokines, which influence macrophage secretion and regulate the immune response, thereby maintaining a healthy balance between the gut microbiota and the host immune system (17).

Gut microbiota is essential for the differentiation and function of resident macrophages. During the activation of macrophages, gut microbiota promotes the development of CD206 expressed macrophages in intestinal muscle layer (20). Prior research has demonstrated that colonic macrophages in specific pathogen-free (SPF) mouse exhibit heightened immune defense, antigen presentation, oxidative phosphorylation, and gene translation in comparison to germ-free (GF) sterile mice. The intestinal microbiota comprehensively influences metabolic processes, the epigenetic regulation of gene expression, host defense mechanisms, and adaptive immunity (21).

Neuro-immune communication between enteric neurons and macrophages induces the rapid tissue protective response to external disturbances. For instance, in response to intestinal bacterial infection, the exogenous sympathetic nerve innervation in the gut is swiftly activated and the norepinephrine is released in the intestinal muscular region. This neurotransmitter mediates signal transduction through β2 adrenergic receptors (β2AR) in the intestine, and then promotes the anti-inflammatory effects of macrophages and enhances the protection effects of the intestinal tissue (22). In the normal state, macrophages also support the enteric nervous system by providing the TGFβ family member bone morphogenetic protein 2 (BMP2) (23), the complement component C1q (24), and the potential cytokine RELMα (22). In turn, neurons support macrophages by providing colony-stimulating factor 1 (CSF1) and influence their differentiation by releasing norepinephrine.

### 2.4 Polarization of intestinal macrophages

The polarization of macrophages refers to the different activation states that macrophages adopt in response to specific environmental signals (**Figure 1**). Macrophages can be activated in two principal ways. One is the classically activated macrophages (CAMφs), also known as the pro-inflammatory (M1) macrophage phenotype, which arises in inflammation environments dominated by TLR and interferon signaling. Two signals *in vitro* could activate these macrophages: interferon-γ (IFN-γ) and lipopolysaccharide (LPS) or other TLR ligands. Promonocytes respond to TLR ligands and acquire the M1 phenotype drove by TLR4 activation and upregulation of nuclear factor-kappa B (NF-κB). Accordingly, these cells produce high levels of pro-inflammatory cytokines, such as TNF, IL-1β, IL-6, IL-12, IL-23, and CCL2, to promote immune responses against bacteria, intracellular pathogens, and tumor cells (25–27).

The second type is alternatively activated macrophages (AA-Mφn), also known as anti-inflammatory (M2) macrophage phenotype. This phenotype is induced by exposure to glucocorticoids, immune complexes, LPS, and Th2 cytokines (such as IL-4, IL-10, and IL-13). Nevertheless, the M1/M2 phenotype does not entirely correspond to the phenotypic subsets of macrophages. Depending on the activating stimulus received, M2 macrophages are classified into four distinct subsets: M2a, M2b, M2c, and M2d. These four subsets also differ in macrophage cell surface markers, secretions, and functions. M2a macrophages are activated by IL-4 and IL-13, which promote the expression of IL-10, TGF-β, CCL17, CCL18, and CCL22. Additionally, these cells increase phagocytic activity, facilitate wound healing and tissue repair, and promote TH2-type cell responses (25, 27). M2b macrophages are activated by immune complexes (IC) and stimulation through TLR or IL-1R, leading to the activation of various transcription factors such as NF-κB, MAPK, and interferon regulatory factor 3, as well as the PI3K-AKT signaling pathway. These cells secrete pro-inflammatory factors, including IL-1β, IL-6, TNF-α, CCL1, and TNF superfamily member 14 (TNFSF14), while also expressing and secreting significant amounts of the anti-inflammatory cytokine IL-10 and low levels of IL-12 (28, 29). M2b macrophages possess potent anti-inflammatory and immunosuppressive effects, ultimately promote infection and tumor progression. Additionally, M2c macrophages are induced by glucocorticoids, IL-10, and TGF-β. These cells secrete high levels of IL-10, TGF-β, CCL16, and CCL18, and are demonstrated with strong capabilities in anti-inflammatory and fibrotic repair. They also play a vital role in the phagocytosis of apoptotic cells (30–32). M2d macrophages considered as TAMs, are induced through co-stimulation by TLR ligands and A2 adenosine receptor (A2R) agonists or IL-6. These cells release IL-10 and VEGF, and promote angiogenesis and tumor progression (33, 34).

In preliminary studies, general macrophages are typically marked by CD63, CD68, and F4/80. Further classification reveals that specific markers for M1 macrophages include CD80, CD86, and iNOS, while particular markers for M2 macrophages include CD163, macrophage mannose receptor (MMR)/CD206, and arginase 1 (Arg1). Furthermore, the expression of particular markers varies among the M2 subtypes. A comparative analysis of the human and mouse macrophage systems has revealed that the unique surface markers of M2a macrophages in humans include CD206, IL-IRa, and IL-IRII. However, in mice, the surface markers of M2a macrophages include Found in inflammatory zone (FIZZ1), YM1/2, and Arg-1 (35). The specific surface markers for M2b macrophages include IL-10R, IL-12R, IL6R, and CD86. Additionally, M2c macrophages exhibit distinct surface marker profiles in human and mouse systems. In humans, M2c macrophage cell surface markers encompass MMR/CD206, TLR-1, and TLR-8. However, in mice, the sole surface marker of M2c macrophages is Argin-1 (36).As for M2d macrophages, their specific markers still need to be thoroughly studied. However, several studies have demonstrated that VEGF, IL-12, and TNF-a characterize the surface markers of M2d at relatively low levels, while IL-10 is present at high levels (37).

Notably, regulatory macrophages (RMφ) represent a distinct macrophage subset defined by their unique phenotypic and functional characteristics. Unlike classical M1 or M2 polarization, RMφ are typically induced by combinatorial stimuli, including TLR ligands (e.g., LPS), high-density immune complexes, and immunomodulatory molecules such as adenosine and prostaglandins. Functionally, RMφ exhibit potent immunosuppressive activity mediated through two primary mechanisms (1): secretion of anti-inflammatory cytokines (e.g., IL-10, TGF-β) that dampen effector T cell responses, and (2) upregulation of co-inhibitory molecules (e.g., PD-L1) to directly suppress T cell activation (38). While RMφ share partial overlap with M2 macrophages in tissue repair functions, their specialized role in immune tolerance, such as promoting Treg expansion and mitigating inflammatory damage, distinguishes them from both pro-inflammatory M1 and pro-repair M2 subsets (39). However, whether RMφ constitute a standalone subtype equivalent to the M1/M2 dichotomy remains debated, largely due to heterogeneity in activation markers (e.g., CD163 vs. CD206 expression) (25) and context-dependent plasticity. Standardized criteria integrating transcriptomic, epigenetic, and functional profiling are required to resolve this classification ambiguity (40).

Macrophage polarization is a dynamic process. There is no absolute distinction between the M1 and M2 phenotypes, and these cells coexist and may even transdifferentiate under specific conditions. In a healthy organism, these various states maintain immune homeostasis. In summary, different macrophage subpopulations play irreplaceable roles in the body. Different forms of activation of macrophages promote or inhibit the development of inflammation, which directly affects the development of inflammation-induced tumors (such as CAC). Therefore, the key to therapies targeting macrophages is to alter their phenotypes without affecting their fundamental physiological functions.

## 3 Macrophage polarization influences the occurrence and progression of colitis-associated colorectal cancer

### 3.1 The roles of M1 and M2 macrophages in intestinal inflammation

**Figure 2:**
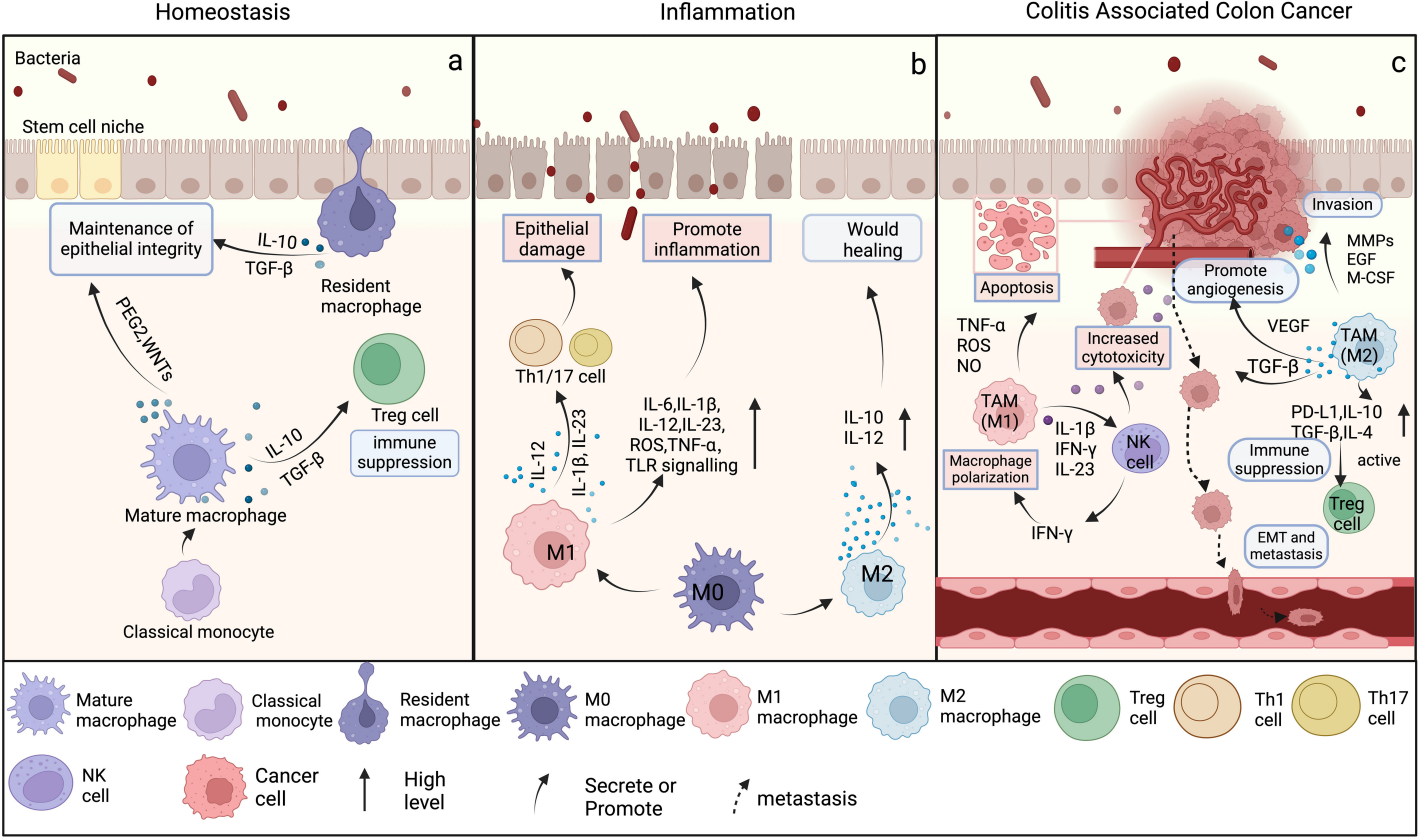
The differentiation and function of macrophages in intestinal homeostasis, inflammation, and colitis associated colon cancer. *(Created in BioRender. yujie*, *(D)* (2025) *https://BioRender.com/n37y529* ) **(A)** In homeostasis, classical monocytes migrate from the bloodstream into the lamina propria, where they differentiate into mature macrophages. These mature macrophages and resident macrophages predominantly secrete low levels of IL-10 and TGF-β to maintain epithelial barrier integrity and immune quiescence. Regulatory T cells (Tregs), dependent on these anti-inflammatory signals, further suppress excessive immune activation to preserve tissue equilibrium. In contrast, IL-6 production, TLR signaling, and iNOS activity remain minimally active under steady-state conditions, becoming robustly upregulated only upon pathogen encounter or tissue injury. **(B)** During intestinal inflammation, mature macrophages predominantly differentiate into the M1 phenotype, secreting pro-inflammatory cytokines and exacerbating epithelial damage. Concurrently, some macrophages differentiate into the M2 phenotype, producing IL-10 and IL-12 to eliminate inflammation and promote wound healing. **(C)** During CAC, M1 TAMs produce a substantial number of pro-inflammatory cytokines, which intensify the inflammatory response of the TME, stimulate the activation of cytotoxic T cells, and augment the capacity of the immune system to eradicate tumors. However, the anti-inflammatory cytokines secreted by M2 TAMs influence tumor progression by inhibiting apoptosis, facilitating invasion, enhancing angiogenesis, and inducing EMT.

Macrophages are critical gatekeepers of intestinal immune homeostasis, and inflammatory bowel disease (IBD) is a direct result of the immune disorder (**Figure 2**). IBD includes ulcerative colitis and Crohn’s disease, both of which are closely linked to immune dysfunction (41). Patients with active IBD and mice models of colitis induced by DSS exhibit increased inflammatory macrophages in the intestinal mucosa (42). These macrophages originate from classical monocytes and secrete large amounts of pro-inflammatory cytokines and chemokines, which facilitate the recruitment and sustenance of pathogenic effector T-cell responses. Cytokines such as GM-CSF and IFN-γ then serve to further augment the M1 phenotype of macrophages, which release even more pro-inflammatory cytokines, including IL-1β, IL-6, IL-2, TGF-β, and TNF. Consequently, this process activates fibroblasts, which in turn induce the production of monocyte chemotactic factors, thereby establishing a positive feedback loop (19, 43). The IL-22 produced by effector T cells drive pro-inflammatory responses in intestinal epithelial cells, which include the release of neutrophil and monocyte chemoattractant molecules, further enhance the recruitment of highly pro-inflammatory cells (44, 45). In multiple mouse models of colitis, inhibition of IL-12/IL-23 p40, IL-23 p19, or IL-23 receptor function significantly suppresses intestinal inflammation by reducing the activation of IL-23 target cells (such as T helper cells 17, innate lymphoid cells 3, neutrophils and natural killer cells) and pro-inflammatory cytokines (46). This indicates that the regulation of macrophages, which produce pro-inflammatory cytokines, is linked to disease susceptibility. Therapies targeting the blockade of pro-inflammatory factors, such as TNF-α inhibitors, have shown promising efficacy in alleviating and treating IBD.

M2 macrophages secrete the anti-inflammatory cytokines IL-10 and low levels of IL-12, and counteract the effects of M1 macrophages (47). They inhibit antigen presentation and serve as potent inhibitors of pro-inflammatory cytokines, chemokines, and inflammasomes, thereby promoting intestinal homeostasis restoration and healing (48). Studies have also found that IL-10 signaling in macrophages contributes to the induction of CD206^+^ regulatory macrophages and therapeutic response to anti-TNF (49). Recent studies have found that *Pediococcus pentosaceus* (*P. pentosaceus*) polarize intestinal macrophages toward the anti-inflammatory M2 phenotype. This shift results in a decreased production of IL-1β, and lead to reduce the levels of reactive oxygen species (ROS), decrease the activation of NF-κB, and lessen apoptosis of intestinal epithelial cells. These effects contribute to the repair of intestinal barriers in juvenile mice with colitis and help in the modulation of the gut microbiota (50).

### 3.2 The roles of M1 and M2 macrophages in colitis-associated colorectal cancer

Macrophage polarization influences the CAC progression (**Figure 2**). CAC refers to colorectal cancer caused by chronic inflammatory diseases, such as IBD (51). M1-TAMs produce substantial quantities of pro-inflammatory cytokines, such as IL-1β, interferon-beta (IFN-β), and IL-23. These cytokines enhance the expression of natural killer (NK) cell-associated proteins, and activate NK cells cytotoxic response against target cells. This activation stimulates NK cells to secrete IFN-γ, which further augments the polarization of M1-type macrophages and promotes the secretion of cytokines that induce apoptosis in tumor cells (52). Moreover, M1 macrophages secrete various cytokines that enhance T-cell activation, cytokine production, proliferation, and differentiation. They also increase the infiltration of neutrophils at the tumor site and contribute to the targeted elimination of tumors by pro-inflammatory neutrophils (53–55). M1 macrophages promote the immune response and inhibit tumor progression through multiple pathways. Kshipra Singh et al. found that the removal of ornithine decarboxylase (ODC) limits the activation of M1 macrophages, while the number of M1 macrophages is restored without affecting M2 macrophages. In the AOM-DSS model, the number and burden of tumors are reduced in Odc^mye^ mice, the mucosal innate immune response is enhanced, and the development of colon tumors is suppressed (56).

In contrast to M1 macrophages, M2 macrophages display anti-inflammatory and pro-tumor functions with more intricate underlying mechanisms. Sun Mi Hong et al. have demonstrated that during the development of CRC, NAMPT is involved in the polarization of M2 macrophages by stabilizing HIF-1α. The elevated levels of HIF-1α facilitate the phosphorylation of STAT3, thereby activating oncogenic signaling pathways that contribute to the progression of CRC (57). In the tumor microenvironment, the M2-TAMs release many other cytokines to encourage tumor invasion, including M‐CSF, MMPs, and EGF. Interestingly, the secretion of M‐CSF could cause TAMs to maintain the M2‐like phenotype, thus forming a circulation that promotes tumor development continuously (58). M2-TAMs secrete IL-10, TGF-β, and PGE2, and promote tumor angiogenesis and tissue remodeling. They also reduce the production of cytotoxic substances such as nitric oxide (NO) and inducible iNOS, thereby inhibiting the activity of tumor-killing cells and weakening the cytotoxic effects of M1 macrophages on tumor cells (59). Additionally, M2-TAMs primarily exert their effects on transformed intestinal epithelial cells, promoting proliferation, inhibiting apoptosis, facilitating invasion, stimulating angiogenesis, inducing epithelial-to-mesenchymal transition (EMT), and enhancing metastasis (60). Accordingly, M2-TAMs can also enhance resistance to CACtherapies. Wei C et al. demonstrated that CCL22 secreted by M2-TAMs in the CRCtumor microenvironment counteracts the antitumor effects of 5-FU by activating the PI3K/AKT pathway (61). The upregulation of PD-1 and PD-L1 by M2-TAMs is a direct consequence of immunosuppression. This could eliminate therapeutic antibodies used for immune checkpoint blockade, and significantly reduce the effect of PD-1-targeted therapies in CAC (62).

### 3.3 The role of macrophage polarization on the progression from colitis to colon cancer

Macrophages primarily influence the progression of IBD to CRC through the secretion of cytokines (63). CAC is often considered a specific subtype of cancer induced by inflammation. Unlike typical colorectal cancer, CAC typically follows a distinct progression characterized by the sequence of “inflamed mucosa - dysplasia - cancer.” (64). Several retrospective studies and meta-analyses have indicated that extensive inflammatory responses in IBD are an independent risk factor for the development of CAC (65).

IBD is characterized by a chronic inflammatory state marked by the disruption of intestinal barrier function, defects in Paneth cells, and alterations in the host microbiota. These shifts can result in an imbalance within the intestinal ecosystem, leading to the formation of a locally inflammatory environment. Prolonged exposure to the intricate environment created by the interplay of various secreted factors and matrix remodeling enzymes that are aberrantly expressed can expedite the progression of colorectal cancer and induce a systemic response that influences the outcome of the disease (66).

M1 macrophages secrete pro-inflammatory cytokines such as IL-1β, IL-6, IL-11, IL-13, IL-23, IL-33, and TNF-α, which trigger inflammatory responses in the gut and contribute to the persistence of inflammation or inadequate resolution in IBD. However, the risk of CRC increases by approximately 2 to 3 times in individuals whose intestines are in a state of chronic inflammation (67). Among these cytokines, TNF-α and IL-1β are particularly significant, as they play a crucial role in transmitting inflammatory signals that lead to or promote the development of CRC. TNF-α is mainly produced by M1 macrophages, and its expression is upregulated in an inflammatory environment, and it play a central role in the pathogenesis of IBD. In addition, TNF-α could activate several signaling pathways, including the NF-κB and MAPK pathways, thereby activating c-Jun N-terminal kinase (JNK) and activator protein 1 (AP-1) (68). The sustained activity of NF-κB and AP-1 promotes the progression of colitis to CRC. As mentioned earlier, IL-1β exerts pro-inflammatory effects by recruiting phagocytes and enhancing Th17 differentiation. Moreover, IL-1β binds to IL-1R1 on the surface of intestinal epithelial cells and activates pro-inflammatory pathways mediated like MAPK/AP-1 or NF-κB that promote cell proliferation and survival, angiogenesis, invasion, and metastasis, ultimately leading to the development of CRC (69).

In chronic colitis, the M2 macrophages play a role in alleviating short-term inflammation via their anti-inflammatory properties. However, in the long term, immunosuppression induced by these cells could contribute to sustained inflammatory responses and accelerate cancer development. The proportion of M2 macrophages in human colon cancer tissue is significantly higher than in healthy or inflamed tissue. The characteristic markers of these macrophages (such as CD206 and Arg1) are highly expressed in cancer tissue, further corroborating the pivotal role of M2 macrophages in transforming inflammation into cancer (70). Additionally, the AOM/DSS-induced CAC mouse model showed a notable increase in the number and proportion of M2 macrophages during the disease progressed from inflammatory to cancer. This change is associated with the immunosuppression and angiogenesis promoted by M2 macrophages, which support the survival and expansion of cancer cells by releasing anti-inflammatory factors such as IL-10 and TGF-β and pro-tumor factors. Furthermore, the pro-tumor factors secreted by M2 macrophages (such as VEGF and IL-6) promote cancer cell proliferation while assisting cancer cells in invading and metastasizing by remodeling the extracellular matrix (such as by secreting MMP-9) (71). M2 macrophages also interact with myeloid-derived suppressor cells (MDSCs) to promote the accumulation of M2 macrophages through the secretion of exosomes, thereby further accelerating the process of carcinogenesis (72). In addition, Peritoneal macrophages serve as critical mediators of CRC peritoneal metastasis, orchestrating tumor progression through multifaceted mechanisms. Specifically, their pro-metastatic capacity is driven by the enrichment of the SPP1^+^ macrophage subset, which enhances tumor cell invasiveness and remodels the ECM through secretion of osteopontin (SPP1) and CXCL12, thereby activating pathways such as the SPP1-CD44/PTGER4 signaling axis (73). Concurrently, these macrophages upregulate the HIF-1α pathway to augment tumor cell glycolytic metabolism, enabling survival within the hypoxic peritoneal niche. These macrophages exhibit a pronounced M2 polarization bias and overexpress immune checkpoint molecules and facilitate immune evasion. Furthermore, peritoneal macrophages engage in a synergistic crosstalk with cancer-associated fibroblasts (CAFs): CAFs secrete CXCL12 and TGF-β to reinforce macrophage M2 polarization, while macrophages reciprocally enhance CAF-mediated ECM remodeling through IL-1β secretion, collectively fostering a pro-metastatic stromal niche (57, 74, 75). Notably, the CXCL12-M2 macrophage axis further amplifies tumor cell resistance to chemotherapeutic agents such as cisplatin, underscoring their role in therapeutic recalcitrance.

In addition, two key genes(COX-2 and NF-κB), which are involved in the inflammatory process, have established a mechanistic link between inflammation and cancer and accelerated the progression of IBD to CAC (76). COX-2 is significantly overexpressed in colorectal tumors, and COX-2-derived PGE2 signaling, which is the downstream of PPARδ pathway, mediates the crosstalk between tumor epithelial cells and macrophages and promotes chronic inflammation and the development of inflammation-related colorectal cancer (77). PGE2, the primary downstream mediator of COX-2, is mainly secreted by intestinal macrophages. It promotes cell proliferation and angiogenesis, inhibits apoptosis, enhances invasiveness, and regulates immunosuppression (78). Furthermore, the transcription factor NF-κB is activated by various carcinogens and growth factors, including microbial flora and pro-oxidants, during inflammatory stimuli. It plays a central role in inflammation and is primarily expressed in cancer. NF-κB appears to be implicated in the recruitment of TAMs (79). In turn, the cytokines produced by activated macrophages could further activate NF-κB and increase the expression of various inflammatory and tumor-promoting cytokines (such as IL-6, IL-1α, and TNF-α) and genes such as BCL-2 and BCL-XL. These molecular interactions provide an opportunity for tumor development (80). In addition to inflammation, several other mechanisms contribute to the development of CAC, including ROS (81), inflammasomes (82), and specific types of cell death, such as pyroptosis and necrosis (83), These mechanisms also play an active role in the progression of CAC.

In summary, macrophages provide new perspectives on controlling the evolution of CRC. Regulating macrophage polarization and inhibiting cytokine secretion could delay the progression from colitis to CRC, and lead to a better prognosis for in the both diseases.

## 4 Factors influencing intestinal macrophage polarization

### 4.1 Metabolic pathways and metabolites

Current studies suggest that multiple metabolic pathways and their metabolites play a significant role in modulating the polarization of TAMs (84). The glycolytic pathway appears to influence the cytokine production processes in M1 and M2 macrophages, with a mechanism closely linked to mitochondrial oxidative phosphorylation. Adding glycolysis inhibitors, such as 2-deoxy-D-glucose or dichloroacetate, during LPS-stimulated M2 differentiation results in significantly reduced IL-10 levels in M2 macrophages compared to the absence of these inhibitors. Conversely, IL-6 production is markedly elevated (85). The glycolytic metabolite lactate, produced by the tumor cells, stabilizes HIF-1α, which in turn induces VEGF expression and promotes M2-like polarization of TAMs (86). M2 macrophages have a greater dependence on ATP produced via the tricarboxylic acid (TCA) cycle. Unlike M1 macrophages, which could compensate for TCA cycle inhibition through alternative metabolic pathways, M2 macrophages depend more on TCA cycle-derived energy. SMYD3, a lysine methyltransferase from the SMYD family, could activate the TCA cycle, and promote ROS generation and upregulate genes associated with the mitochondrial respiratory chain complex. This activity facilitates the repolarization of M1 macrophages toward the M2 phenotype (87). Studies have found that an increase in the number of functional mitochondria enhances the ability of M2 macrophages to undergo remodeling (88). In the TCA cycle, α-ketoglutarate and succinate play pivotal roles in macrophage polarization. α-Ketoglutarate is a critical metabolite that induces macrophage polarization toward the M2 phenotype, whereas succinate enhances aerobic glycolysis and ROS production, and drive macrophage polarization toward the M1 phenotype (89). This suggests that the balance between α-ketoglutarate and succinate is crucial for stabilizing macrophage polarization states. A disruption in their ratio could drive macrophages to shift toward either the M1 or M2 phenotype. Notably, fatty acid oxidation (FAO) also serves as a significant energy source for the polarization of macrophages toward the M2 phenotype. Upregulation of the FAO rate-limiting enzyme, carnitine palmitoyl transferase 1a (Cpt1a), enhances fatty acid metabolism in macrophages and promotes M2 polarization, thereby accelerating the progression of CAC (90).

### 4.2 Cytokines

TAMs are macrophages in the TME. In CAC, signals in the TME, such as cytokines, chemokines, growth factors, and matrix metalloproteinases, influence the metabolic reprogramming of macrophages, and cause TAMs polarization and exhibit different phenotypes and functions (91). The polarized macrophages mainly affect tumor survival, metastasis, and prognosis. TNF, IL-6, IL-8, and TGF are secreted by macrophages. The function of these cytokines is dynamic and multifaceted in the context of the immune microenvironment of inflammatory bowel disease and colorectal cancer. This is due to the close relationship between the functional conversion of these cytokines and the dynamic changes in cell interactions and signaling networks in the microenvironment (**Table 1**).

**Table 1:** Comparison of the dual role of cytokines in inflammatory bowel disease and colorectal cancer.

Cytokine	Inflammatory Bowel Disease	Colorectal Cancer
**TNF-α**	**Pro-inflammatory:** - Activates M1 macrophages (95) - Enhances neutrophil infiltration and intestinal barrier disruption	**Dual Role:** - Early phase: Suppresses tumor growth - Late phase: Activates M2 macrophages (97), promotes EMT and vascular leakage (pro-metastatic)
**IL-6**	**Pro-inflammatory:** - Activates M1 macrophages (99) - Induces hepatocyte production of C-reactive protein (CRP), enhancing acute-phase response (177)	**Pro-tumorigenic:** - Activates M2 macrophages, promotes tumor cell proliferation and immune evasion - Induces angiogenesis (VEGF) and pre-metastatic niche formation (102)
**TGF-β**	**Anti-inflammatory:** - Activates M2 polarization and IL-10 secretion to resolve inflammation (103) - Enhances mucosal repair	**Pro-tumorigenic:** - Activates M2 macrophages, drives EMT and ECM remodeling (pro-invasive) - Expands Tregs to suppress anti-tumor immunity - Drives angiogenesis (VEGF/FGF2) (107)
**IL-8**	**Pro-inflammatory:** - Activates M1 macrophages - Recruits neutrophils, releasing ROS to exacerbate inflammation (114)	**Pro-tumorigenic:** - Activates M2 macrophages - Drives angiogenesis (VEGF/FGF2) -Modulates metabolism, enhance chemotherapy and immune checkpoint inhibitors (113)

TNF-α-mediated pro-inflammatory pathways play a key role in promoting cancer development (92). TNF is a homotrimer that binds to two different receptors on the cell surface: TNF receptor 1 (TNFR1, also known as the p55 receptor) and TNF receptor 2 (TNFR2, also known as the p75 receptor) (93). Both in the DSS-induced colitis model and AOM/DSS-induced CAC model, TNFR2 is highly expressed in the intestinal epithelium, and promoting the activation of NF-κB and other signaling pathways and facilitating cell survival. This also leads to the upregulation of myosin light chain kinase, releasing pro-tumor cytokines and disrupting tight junctions (94). During acute or chronic colitis, TNF-α is primarily secreted by activated macrophages, T cells, and epithelial cells, with its elevated expression predominantly associated with M1-polarized macrophages (95). In the early phases of intestinal inflammation, M1 macrophages contribute to pathogen clearance and necrotic cell removal, thereby facilitating localized inflammatory responses and immune cell recruitment. However, prolonged exposure to TNF-α can intensify local inflammation and tissue injury by activating downstream signaling pathways such as NF-κB and MAPK, which amplify the inflammatory cascade (96). Correspondingly, in the context of CAC, TNF-α exerts a significant influence within the tumor microenvironment. Initially, TNF-α contributes to anti-tumor immunity. However, chronic inflammation and sustained TNF-α signaling lead to DNA damage and genetic mutations. At the same time, TNF-α regulates macrophage polarization toward the M2 phenotype and promotes tumor progression and metastasis by activating complex cytokine networks (97). This dual role of TNF-α underscores its complex regulatory functions across different pathological conditions and provides a foundation for targeted therapies aimed at modulating TNF-α signaling (98).

IL-6 is an essential mediator of inflammation and immunity (99). It binds to the membrane-bound IL-6 receptor (IL-6R) on target cells and transduces signals through the heterodimer complex formed with glycoprotein 130 (gp130). In inflammatory environment, Gp130 is a shared receptor chain of the IL-6 family and an effective inducer of STAT3 activation, which drives macrophage polarization toward an M1 phenotype (100). These M1 macrophages secrete pro-inflammatory cytokines, such as IL-1β and TNF-α, that are essential for pathogen clearance and the elimination of damaged cells during the initial phases of the inflammatory response. Nevertheless, prolonged or recurrent inflammation may result in an overactive M1 response, thereby exacerbating tissue damage and perpetuating chronic inflammatory conditions (99). However, in CAC, IL-6 assumes a tumor-promoting role. In this setting, persistent IL-6 signaling leads to continual activation of STAT3, which not only supports cancer cell proliferation and survival but also reconditions the immune microenvironment (101). This reprogramming favors a shift in macrophage polarization toward the M2 phenotype, characterized by the production of anti-inflammatory cytokines and growth factors such as IL-10 and TGF-β. These factors suppress effective antitumor immunity and facilitate tumor invasion and metastasis (102). Thus, while IL-6 contributes to host defense in colitis by enhancing an M1-mediated pro-inflammatory response, its sustained expression in a chronic inflammatory milieu can promote macrophage reprogramming toward an M2 phenotype, ultimately altering the local immune landscape to support tumor progression.

TGF-β, primarily produced by macrophages, is a pivotal regulator of tissue wound healing and carcinogenesis. In intestinal inflammation, TGF-β exerts immunosuppressive effects by attenuating pro-inflammatory functions of M1-polarized macrophages, such as ROS and NO production. Simultaneously, it promotes macrophage polarization toward an M2 phenotype characterized by anti-inflammatory mediator secretion, including IL-10 and TGF-β itself. The resulting M2-skewed microenvironment suppresses CD8^+^ T cell cytotoxicity and dampens excessive inflammatory responses, thereby maintaining immune homeostasis and tissue integrity (103). Mechanistically, TGF-β coordinates crosstalk between T cells and macrophages to regulate localized inflammation and mitigate tissue damage. However, chronic or hyperactivated TGF-β signaling may paradoxically induce immune evasion by suppressing effector immune responses, exacerbating persistent inflammation and accelerating chronic intestinal pathology. In CAC, TGF-β exhibits heightened functional complexity. On the one hand, TGF-β drives M2 macrophage polarization to facilitate tumor immune evasion and suppress antitumor immunity (104). Through both canonical SMAD-dependent signaling and non-canonical pathways (e.g., ERK and PI3K/AKT), TGF-β enhances tumor cell invasiveness and recruits M2 macrophages to remodel the ECM, establishing a pre-metastatic niche (74, 105, 106). Additionally, M2 macrophages secrete pro-angiogenic factors such as VEGF and FGF2, promoting tumor vascularization and metastatic dissemination (107). Thus, the dual roles of TGF-β in colitis and colorectal cancer underscore the need for stage-specific therapeutic strategies to achieve precise clinical intervention.

IL-8 plays a pivotal role in macrophage polarization, primarily through regulating chemokine secretion and intercellular signaling. IL-8 is primarily secreted by intestinal epithelial cells, macrophages, and neutrophils in enteritis (108). It acts by binding to CXCR1/CXCR2 receptors, thereby recruiting neutrophils and monocytes to the sites of inflammation (109, 110). During the early stages of acute enteritis, IL-8-mediated neutrophil infiltration aids in pathogen clearance. However, prolonged high expression of IL-8 leads to macrophage polarization toward the M1 phenotype and inhibits the anti-inflammatory function of M2 macrophages. Additionally, IL-8 enhances the pro-inflammatory functions of M1 macrophages, such as the secretion of IL-1β and TNF-α, through the activation of the NF-κB pathway. This creates a positive feedback loop that further exacerbates intestinal inflammatory injury (111). In CRC, IL-8 is secreted by TAMs and cancer cells and promotes tumor angiogenesis and pre-metastatic microenvironment formation by inducing the expression of VEGF and FGF2. In addition, IL-8 interacted with CXCR2^+^ myeloid-derived suppressor cells (MDSCs) to enhance their immunosuppressive function and promote tumor immune escape. Not only that, IL-8 enhances the resistance of tumor cells and macrophages to chemotherapy and immune checkpoint inhibitors by modulating their metabolism (e.g. glycolysis and fatty acid oxidation). For example, IL-8 can impair the efficacy of anti-PD-1 therapy by upregulating PD-L1 expression (112, 113). This bidirectional influence may be associated with the differentiation and functional heterogeneity of TAMs (114).

### 4.3 Signaling pathway

The main signaling pathways that influence the reprogramming of TAMs include the NF-kB signaling pathway and Janus kinase (JAK)/STAT signaling pathway.

NF-κB regulates a variety of physiological processes, including immune and inflammatory responses. Transcription facilitated by this pathway represents a principal regulatory factor affecting the expression of multiple cytokines in the TEM (115, 116). It is postulated that aberrant activation of NF-κB may play a role in the progression of CAC. NF-κB positively influences the processes of M2 polarization and tumor progression (117). The aberrant activation of NF-κB is considered a contributing factor in the progression of CRC. Evidence suggests that it plays a positive role in M2 macrophage polarization and tumor progression (117). In the mouse AOM\DSS model, Michael Karin and colleagues have reported two signaling pathways that lead to the activation of NF-κB. The first is the classical pathway, activated by TNF-α, IL-1, LPS, CD40 ligand (CD40L), and to a lesser extent, by light-sensitive molecules α/β (LT α/β) and Blys/BAFF. This pathway is mediated through the IKK (IκB kinase) complex, which consists of three subunits: the catalytic subunits IKK-α and IKK-β, and the regulatory subunit IKK-γ. The second is the alternative pathway, which can be activated by LT α/β, CD40L, and Blys/BAFF but not by TNF-α, IL-1, and LPS. Activation of this pathway depends on the IKK-α homodimer, which induces the processing of p100 and the nuclear translocation of the RelB-p52 dimer. These two pathways are essential for activating innate immunity and inflammation and inhibiting apoptosis or the development of secondary lymphoid organs, B cell maturation, and adaptive humoral immunity (118). In the AOM/DSS-induced murine CAC model, Th17-related cytokines, including IL-17A, IL-21, IL-22, TNF-α, and IL-6, are produced by tumor-infiltrating lymphocytes (TIL), which could activate the STAT3/NF-κB pathway, thereby promoting CAC cell proliferation and accelerating tumor progression (92). Moreover, the level of NF-κB activity also influences the balance of Treg differentiation, thereby modulating immune tolerance and inflammatory responses (119). In the tumor microenvironment, fibroblasts, particularly CAFs, are frequently subject to sustained NF-κB activation. Consequently, activated NF-κB drives these fibroblasts to secrete a range of cytokines and chemokines, such as IL-6, IL-8, and MMPs. These factors not only remodel the ECM, thereby creating conditions conducive to tumor cell invasion and metastasis, but also attract and activate various immune cells, including T cells (120). Furthermore, through the NF-κB-mediated cytokine network, fibroblasts engage in complex signaling interactions with both tumor cells and immune cells, ultimately regulating local inflammation and promoting immune evasion.

STAT3, situated at the convergence of multiple signaling pathways, is indispensable for the survival of intestinal epithelial cells and the preservation of mucosal integrity. It functions as a transcriptional mediator of oncogenic signaling and plays a pivotal role in the polarization of macrophages (121). Upon activation, STAT3 induces the transcription of Bclns and Mclnsc two key antiscriptionE proteins that support macrophage viabilityeionE.DATA ot in environments characterized by chronic inflammation or tumorigenesis, where preventing apoptosis is crucial for sustained cell survival. A considerable body of research has demonstrated that the JAK/STAT3 axis contributes to the preferential polarization of macrophages toward an M2-like phenotype across diverse pathological settings. In this context, SOCS proteins, especially SOCS3, operate as critical downstream modulators by exerting negative feedback on the pathway. By inhibiting JAK kinases, SOCS3 serves to prevent uncontrolled STAT3 activation, thereby preserving immune equilibrium and restraining excessive M2 polarization (122). Clinical studies have revealed that CRC patients with positive expression of JAK1 and STAT3 proteins exhibit significantly reduced survival rates compared to those with negative expression of these proteins. Moreover, this positive expression is linked to increased tumor infiltration and metastasis (123). In the AOM/DSS-induced CAC mouse model, it has been observed that phosphorylation and activation of STAT3 directly affect cell cycle regulators, promoting intestinal epithelial cell survival and leading to excessive proliferation. Furthermore, activated STAT3 could enhance NF-κB activity. In the AOM/DSS-induced CAC mouse model, IL-13 produced by NKT cells induces the polarization of some macrophages to the M2 phenotype. This leads to the production of a large amounts of IL-6 by TAMs, thereby promoting cancer progression (124). In both acute and chronic enteritis, STAT3 activation is associated with an increase in T cell proliferation and activation—most notably among Th17 cells—thus amplifying the inflammatory response (125). While such activation is beneficial for combating infections and repairing tissue damage, its dysregulation may result in excessive inflammation, potentially leading to intestinal injury or chronic inflammatory conditions. Conversely, STAT3 also plays a role in immune tolerance by influencing the differentiation of regulatory T cells, thereby averting autoimmunity in the gut.

### 4.4 Microbiota

In addition to the factors mentioned above, the role of gut microbiota in colorectal tumors has received increasing attention from researchers in recent years (126). The gut microbiota is a vital regulator of the inflammatory potential of intestinal macrophages. Studies have found that after 15 consecutive colonizations of *Escherichia coli 541*, M2 macrophages secrete IL-10, providing protection against intestinal damage, alleviating intestinal inflammation, and limiting the progression of CAC (127). Specific microbial species, such as those representing a dysbiotic gut microbiota (*Atopobium vaginae*, *Selenomonas* sp*utigena*, and *Faecalibacterium prausnitzii*), could recruit B cells and macrophages to activate immune responses specific to CAC. This promotes the M2b polarization induced by the fecal microbiota and enhances the pro-tumor activity of TAMs *in vivo (* 128). It can be observed that enhancing the colonization of certain microbial communities or inhibiting specific microbiota could serve as a strategy for immune therapy in CAC.

## 5 Immunotherapy targeting macrophages in CAC

In summary, tumor-associated macrophages play a role in coordinating angiogenesis, extracellular matrix remodeling, tumor cell proliferation, metastasis, and immune suppression. The cytokines secreted by polarized macrophages influence the activation of critical molecules in classic cancer pathways, suppress innate and adaptive immune responses, and play a crucial role in the antitumor activity of chemotherapy, radiotherapy, and monoclonal antibodies (mAb) (129). M2-like TAMs are involved in various immune-suppressive processes in inflammatory bowel cancer and contribute to resistance in immune checkpoint therapies and CAR-T cell treatments. Macrophage-centered therapeutic strategies have garnered increasing attention. Targeting macrophages can help rebalance the tumor microenvironment from a pro-tumor immune landscape to an anti-tumor immune environment and synergize with T-cell-enhancing drugs (such as checkpoint inhibitors) to combat cancer (130).Therapeutic approaches include blocking the sustained pro-tumor effects of M2 macrophages and harnessing the anti-tumor potential of M1 macrophages. Targeting TAMs for cancer therapy has two main directions (131) (132): preventing macrophage recruitment and regulating TAM polarization (**Table 2**).

**Table 2:** Treatment strategy for reprogramming TAMs in CAC.

Category		Target	drug	CAC mouse model	CAC patients	Clinical status	Mechanism	Combined therapy	References
**Targeting** **recruitment**		CSF-1/CSF-1R	Pexidartinib	Significantly reduces tumor burden and the number of M2 cells	Patients with high CSF1R expression have a poorer prognosis.	Durvalumab combined with Pexidartinib for treating CAC in phase I studies.	Blocks the CSF-1 signaling pathway; Reduces the number of TAMs; promotes the polarization of TAMs to M2	Pexidartinib +Durvalumab	(178, 179) (135).
		CCL2\ CCR2	RS504393 RS102895	Significantly reduced infiltration of TAMs in tumors	Reduced infiltration of immunosuppressive cells in the primary tumor	–	Promotes the infiltration of macrophages and the differentiation of M2-TAMs	RS504393/ RS102895 +anti-PD-1	(138, 139)
		CXCL12 /CXCR4	LY2510924	Inhibits recruitment of TAMs and reduces M2 phenotype	Mitigates tumor metastasis	phase I	Inhibits the CXCL12/CXCR4 signaling axis; reduces the accumulation of M2-TAM; suppresses tumor growth, invasion, and metastasis.	LY2510924 +anti-PD-1	(143)
**Regulating TAM polarization**	Inhibits M2 Polarize	H2-R	Cimetidine	Reducing tumor growth by modulating the immune environment	Potential benefit to patients	–	Inhibits histamine-mediated immunosuppression; reduces M2 macrophages; enhances; immune responses	Cimetidine +5-fluorouracil	(151) (180).
		VEGF	Bevacizumab	It inhibits angiogenesis and reduces tumor growth.	Combination with chemotherapy improves survival in some patients.	Phase III	Inhibits angiogenesis; Inhibits the accumulation of M2 cells in the TME	Bevacizumab + 0xaliplatin + Fluoropyrimidine	(147, 181)
		TNF	Etanercept	Tumor-associated inflammation is reduced	Improved survival rates in some patients	Phase III	Inhibits the pro-inflammatory effects of TNF-α; aggravates tumor-associated inflammation	Etanercept +anti-CTLA-4 +anti-PD-1	(182) (183).
		IL-1β	Canakinumab	Tumor-associated inflammation and tumor burden were significantly reduced	Its potential for preventing CAC is being investigated.	Phase III	Inhibits tumor-related inflammation; reduces the tendency to polarize toward M2 and the cancer risk.	–	(184).
		IL-6	Tocilizumab	It effectively suppresses inflammation-induced carcinogenesis.	Shows benefits in some CRC patients	Phase Ib	Inhibits inflammation by blocking the IL-6 signal; reduces the proportion of M2 macrophages.	Tocilizumab +anti-CTLA-4 +anti-PD-1	(185) (186).
	Promotes M1 Polarize	HDAC	Tucidinostat	Induces M1-type polarization and reduces tumor growth in mice	Enhances immune cell function in the TME	Phase III	Epigenetic regulation polarizes TAMs from M2 to M1; restores T cell function.	Tucidinostat +anti-PD-L1	(187, 188)
		TLR	IMO-2125	The antitumor effect of TAMs was effectively activated	A combination of PD-1 inhibitors is being evaluated	Phase Ib	Promotes TAMs polarization toward M1; enhances antitumor immunity.	–	(176)

### 5.1 Preventing macrophage recruitment in TME

The focus of immunotherapy strategies targeting macrophage recruitment in CAC is primarily on the blocking of key chemokine and chemokine receptor pathways, such as CCL2/CCR2, CSF1/CSF1R, and CXCL12/CXCR4. These strategies have been shown to improve the tumor microenvironment and inhibit tumor progression by reducing macrophage recruitment.

#### 5.1.1 CSF1/CSF1R

CSF1 and its receptor play a central role in the differentiation and survival of the mononuclear phagocyte system and TAMs (133). TAMs stimulated by CSF1 secrete additional CSF1 through paracrine signaling between macrophages and tumor cells, and enhance the invasive properties of tumor cells (134). Because macrophages have a great dependence on CSF1R signaling, CSF1R has become a critical target for selectively depleting macrophages. The CSF1/CSF1R axis is essential for the survival and differentiation of M2-TAMs in CRC. Consequently, targeting CSF1R presents a promising therapeutic approach to reduce M2-TAMs presence and enhance antitumor immunity (135). Recent studies have found that targeting CSF1R could also directly inhibit CRC development and metastasis through the miR-34a/CSF1R pathway while overcoming resistance to 5-FU treatment (136). In order to achieve optimal efficacy, the concurrent administration of immune checkpoint inhibitors (e.g. PD-1/PD-L1 or CTLA-4 antibodies) can disrupt the state of immune tolerance within the tumor immune microenvironment. This enhances the synergy between T cells and macrophages, as well as promoting ‘immune remodeling’ of the tumor immune microenvironment. Consequently, the tumor becomes more susceptible to attack by the immune system. Furthermore, the combination of CSF1R inhibitors and anti-VEGF therapy not only assists in the reduction of immunosuppressive TAM recruitment, but also enhances the efficacy of immunotherapy by promoting the infiltration of immune cells.

#### 5.1.2 CCL2/CCR2

CCL2 is highly expressed in many tumors. By binding to its receptor CCR2, CCL2 mainly promotes the recruitment of monocytes and pro-macrophages to the tumor microenvironment. In addition, local conditions induce their differentiation into TAMs (137). These TAMs typically have an M2 phenotype and secrete immunosuppressive factors that promote tumor growth, angiogenesis and matrix remodeling. In a series of studies utilizing mouse models of colorectal cancer, the employment of CCR2 antagonists (e.g., RS504393, RS102895) or the suppression of the CCL2/CCR2 axis has been demonstrated to result in a substantial reduction in the infiltration of TAMs within tumors (138, 139). CCR2 antagonists have been demonstrated to possess limited inhibitory effects on tumor growth in isolation. However, when employed in conjunction with other therapeutic modalities, such as ICB and chemotherapeutic agents, they have been observed to enhance the tumor microenvironment and stimulate an anti-tumor immune response. A number of studies have reported that the incorporation of CCR2 antagonists into combination PD-1/PD-L1 antibody therapy has resulted in increased T cell infiltration, decreased immunosuppressive cells, and a significantly superior overall therapeutic effect in comparison to monotherapy (140).

#### 5.1.3 CXCL12/CXCR4

The CXCL12/CXCR4 signaling axis is integral to both the recruitment and polarization of macrophages in the TME. By driving the accumulation of TAMs and promoting an M2 phenotype, this pathway plays a key role in facilitating tumor growth, invasion, and metastasis (141). Recent studies have shown that the expression level of CXCR4 in primary tumors correlates with the response of patients with metastatic colorectal cancer (mCRC) to first-line chemotherapy (142). Clinical trials indicate that the CXCR4 inhibitor LY2510924 targets the CXCL12-CXCR4 axis, exhibits a favorable safety profile, and is well tolerated in patients with colorectal cancer, pancreatic cancer, and other solid tumors (143). In addition, inhibiting CXCL12 has been found to reduce the infiltration of immunosuppressive cells, such as Treg cells and M2 macrophages, thereby enhancing the efficacy of PD-1/PD-L1 inhibitors (144). However, to date, no preclinical or clinical studies have reported the use of CXCR4 monoclonal antibodies in cancer treatment. Moreover, targeting CXCR4 alone is insufficient to counteract the pro-metastatic effects mediated by CXCL12. In contrast, combining a CXCL12 antagonist with immune checkpoint inhibitors has been shown to achieve better therapeutic outcomes.

### 5.2 Regulating TAM polarization

In CAC, the tumor microenvironment frequently manifests an immunosuppressive state, wherein macrophages predominantly exhibit M2 phenotype, thus facilitating tumor proliferation and metastasis. Consequently, the induction of the differentiation and reprogramming of macrophages to the M1 phenotype has emerged as a pivotal strategy to enhance anti-tumor immunity. The targeting of macrophages in therapy for CAC has been demonstrated to enhance the tumor microenvironment and augment the anti-tumor immune response by reducing the formation of immunosuppressive M2 TAMs and promoting the conversion of macrophages to the M1 phenotype, which has anti-tumor activity. Therefore, we categorized this critical strategy for enhancing anti-tumor immunity into two key approaches: the inhibition of M2 macrophage polarization and the promotion of M1 Macrophage Polarization

#### 5.2.1 Inhibition of M2 macrophage polarization

##### 5.2.1.1 VEGF

VEGF, a member of the growth factor family, plays a crucial role in angiogenesis and creates a favorable environment for tumor growth and metastasis (145). In clinical models, the angiopoietin-2 (Ang-2)/VEGF bispecific antibody exhibits significant antitumor activity and reprograms TAMs from the M2 protumor phenotype to the M1 antitumor phenotype (146). Min AKT et al. found that compared to the normal mucosa of CAC patients, there is a significant increase in the population of M2-TAMs and the expression of VEGFR2 in tumors. Cytokine-induced M2 macrophages *in vitro* produce TGF-β1 through the VEGF/VEGFR2 signaling pathway. This suggests that anti-VEGFR2 treatment could control the immune suppressive function of M2-TAMs in CAC, thereby enhancing the efficacy of immunotherapy (147).

##### 5.2.1.2 Histamine

Both CAC and CRC exhibit marked elevation of histamine within the tumor microenvironment, a feature linked to macrophage-driven immunosuppression (148). Histamine modulates immune responses by engaging macrophage receptors HRH1 and HRH2. After this engagement, there is activation of distinct pathways, and upregulation of M2 markers such as Arg1, IL-10, and CD206. In concert with IL-4/IL-13 signaling, this drives epigenetic remodeling at M2-associated loci, further reinforcing the M2 phenotype (149, 150). The consequent upregulation of VISTA (V-domain Ig suppressor of T cell activation) on histamine-primed M2-TAMs directly impairs T cell effector functions through suppression of TCR signaling pathways (e.g., Lck-ZAP70 phosphorylation) and expansion of Tregs, thereby fostering tumor progression and conferring resistance to immune checkpoint inhibitors. Critically, preclinical models demonstrate that pharmacological blockade of histamine signaling via HRH1/HRH2 antagonists (e.g., loratadine or famotidine) reprograms M2-TAM activation states and rescues antitumor T cell activity, ultimately resensitizing tumors to immunotherapy by disrupting the histamine-VISTA immunosuppressive axis (151).

##### 5.2.1.3 TNF-α

TNF is indispensable for the reprogramming of TAMs. In inflammatory bowel disease, TNF-α has been shown to block the expression of M2-related genes in macrophages and polarize them away from the immunosuppressive M2 phenotype. Studies have demonstrated that a reduction in TNF or a loss of type I TNF receptor signaling leads to increased M2 mRNA expression (152). TNF-α monoclonal antibodies (e.g. Infliximab and Adalimumab) have been extensively utilized in clinical practice to reduce inflammatory responses. In the treatment of intestinal cancer, some studies have concentrated on the combination of TNF inhibitors and immune checkpoint inhibitors (e.g. PD-1/PD-L1 antibodies), given the pro-M2 effect of TNF-α in the tumor microenvironment. This strategy aims to inhibit tumor-promoting inflammation, activate anti-tumor immunity, improve T cell function and remodel the tumor immune microenvironment.

##### 5.2.1.4 PSGL-1

P-selectin glycoprotein ligand-1 (PSGL-1) is widely expressed on hematopoietic-derived cells and serves as a ligand for all selectins (P-, L-, and E-selectins) (153). PSGL-1 binds to chemokines and activates integrins, acting as a negative regulator of T cell function, and is expressed at high levels in TAMs (154). In tumor cells, platelets bind to PSGL-1 expressed on TAMs via P-selectin (CD62P), activating the JNK/STAT1 pathway and the C5a/C5aR1 axis. This results in the differentiation of TAMs into M2 cells, which promote tumor progression, induce immune tolerance and increase tolerance to immunotherapeutic drugs (154). Studies have found that inhibiting the C5a/C5aR1 axis or PSGL-1 significantly reduces the growth of CAC (155). PSGL-1 is involved in reprogramming TAMs and regulating T-cell biology, suggesting that it could serve as a potential drug target for cancer therapy (154).

##### 5.2.1.5 MicroRNA

MicroRNAs are endogenous small non-coding RNAs, typically 18 to 25 nucleotides long, that regulate gene expression by modulating gene transcription and translation (156). MicroRNAs play a critical role in macrophage activation, polarization, tissue infiltration, and the resolution of inflammation (157). By modulating signaling pathways such as NF-κB and STAT3, specific miRNAs can influence the balance between pro-inflammatory M1 and anti-inflammatory M2 macrophage phenotypes, thereby affecting both the inflammatory response and anti-tumor immunity (158). Studies have found that IL-16β drives the secretion of G-MDSC-derived exosomal miR-193-5p, promotes the differentiation of M-MDSCs into M2 macrophages and facilitates the progression of CAC through the STAT3 signaling pathway (72). This suggests that combining drugs that inhibit STAT3 signaling with those that block miR-193-5p secretion could provide an effective therapeutic strategy for CRC. Baer C et al. found that a deficiency of the microRNA processing enzyme DICER in TAMs promotes M1-type polarization, and reduces the immunosuppressive capabilities of TAM. This shift enhances the recruitment of activated cytotoxic T lymphocytes (CTLs) to the tumor, enabling complete tumor eradication when combined with PD-1 checkpoint blockade (159).

##### 5.2.1.6 IL-6

IL-6 exerts a profound influence on macrophage polarization and function through activation of the STAT3 signaling pathway. Within the TME, IL-6 predominantly drives macrophages toward an M2 phenotype, a state that is intimately linked to immunosuppression and the facilitation of tumor growth and metastasis (160). Experimental studies reveal that mice with disrupted IL-6/gp130/STAT3 signaling develop more severe colitis, along with marked epithelial damage and ulceration upon AOM/DSS exposure, compared with wild-type counterparts. Interestingly, these mice exhibit a reduced tumor burden—characterized by smaller and less frequent tumors (161). That may be partly attributed to a diminished presence of tumor-promoting M2 macrophages and an altered inflammatory milieu within the TME, ultimately impeding tumor progression. Blocking IL-6 could enhance ICB-induced antitumor therapy, such as by improving the efficacy of anti-CTLA-4 treatment in preclinical models, reducing autoimmune responses, increasing CD4^+^ and CD8^+^ effector T cells, and decreasing MDSCs and macrophages in the TME. The combination of IL-6 blockade and ICB allows for the decoupling of autoimmunity from antitumor immunity, and offer a novel approach for immunotherapy in CAC and the management of treatment-related complications (162).

##### 5.2.1.7 IL-1β

IL-1β is a potent activator of the NF-κB signaling pathway and plays a crucial role in modulating the TME. It regulates macrophage phenotype switching through both paracrine and autocrine mechanisms. IL-1β has been shown to promote macrophage polarization toward the pro-tumor M2 phenotype, while simultaneously inhibiting the anti-tumor activity of the M1 phenotype (27, 163). Elevated IL-1β levels and expression within the TME are associated with resistance to various anticancer therapies, including cytotoxic agents and immunotherapies, ultimately leading to reduced survival rates (164). By inhibiting IL-1β signaling, these inhibitors mitigate the immune suppressive environment that typically promotes M2 macrophage polarization, which is associated with tumor progression and immune evasion (165). Targeting IL-1β in immunotherapy is an area of active research. For instance, IL-1β monoclonal antibodies, such as canakinumab, have been evaluated in clinical trials for their efficacy in multiple cancers (166). However, the therapeutic effect of IL-1β inhibitors alone appears limited and may require combination with other immunotherapeutic approaches to enhance their anti-tumor activity. Notably, combining IL-1β inhibition with PD-1 blockade has shown a synergistic effect in a non-small cell lung cancer (NSCLC) mouse model, significantly suppressing tumor progression (167). These findings suggest that IL-1β-targeted immunotherapy, particularly when used in combination with other immune therapies, could provide a promising strategy to improve the efficacy of cancer treatments.

#### 5.2.2 Promotion to M1 macrophage polarization

##### 5.2.2.1 HDAC

Histone deacetylase (HDAC) regulates cell proliferation and survival. Notably, Class IIa HDACs modulate immune functions by influencing immune responses, chemokine expression, and the production of complement pathway components (168). Specifically, HDAC4 attenuates the expression of M1 macrophage markers, via modulation of the NF-κF signaling pathway, while concurrently promoting STAT6tingtly,E M2 polarization (169). Recent studies have demonstrated that the Class IIa HDAC inhibitor TMP195 can modulate macrophage dynamics by reducing the overall macrophage population through polarization reprogramming, thereby increasing the proportion of pro-inflammatory M1 macrophages and the secretion of inflammatory cytokines (170). In murine models, the combination of TMP195 with anti-PD-1 therapy significantly reduced tumor burden in both carcinomatous adenomas and subcutaneous tumors, while concurrently enhancing the efficacy of PD-1 blockade (171, 172). These findings suggest that a combinatorial immunotherapeutic strategy integrating HDAC inhibition with immune checkpoint blockade may offer a promising approach for the treatment of carcinomatous adenomas.

##### 5.2.2.2 TLRs

TLR is a crucial pathogen recognition receptor that is expressed by immune system cells. Activation of TLR3 inhibits the co-stimulatory inhibitory receptor Tim-3, enhances antigen uptake and T cell capacity, and inhibits the polarization of M2a and M2c subtypes; then the number of M1 macrophages have significantly elevated and inhibit tumor growth (173). Similarly, TLR9 plays a pivotal role in regulating macrophage function, particularly in the context of inflammatory and immune responses. It has been demonstrated that TLR9 regulates the production of pro-inflammatory cytokines (such as IL-1β, TNF-α, and IL-10) in macrophages (174). The activation could result in various pathological conditions, including the aggregation of macrophages and excessive cytokine production caused by chronic stress. These changes illustrate the involvement of TLR9 in amplifying the inflammatory response through macrophages (175). In addition, combining anti-CTLA-4, anti-PD-1, or anti-PD-L1 therapies with TLR9 agonists may enhance treatment efficacy (176)

## 6 Prospect

Macrophages are essential to intestinal immunity, performing diverse functions crucial for maintaining gut homeostasis. Research on the polarization of TAMs has provided more precise insights into the role of macrophages in the development of CAC. The molecular mechanisms by which TAMs reprogramming promotes the progression of IBD to CAC remain unclear. It is also worth exploring how M2 macrophages shift from an anti-inflammatory role in IBD to a pro-tumorigenic role in CAC. Most studies report that the presence of infiltrative TAMs may be positively correlated with the pathological grading of CAC patients. Therefore, our review focuses on how TAMs polarization influences the progression of CAC by modulating ECM remodeling, tumor metabolism, angiogenesis, and the tumor microenvironment.

In summary, substantial evidence indicates that M2-type TAMs programming is associated with poor prognosis in CAC. Antagonizing M2 phenotype programming at the molecular level can reprogram more TAMs to the M1 phenotype, which counteracts immune resistance and enhances anticancer drug efficacy. The progress of mechanism research and targeting drugs is highly anticipated, as it could lead to identifying new therapeutic targets for CAC. While some targets (e.g., VEGF, CSF-1R, IL-6) have demonstrated preliminary efficacy in preclinical studies and early-phase clinical trials, several major challenges must be addressed to advance their therapeutic potential. First, the inherent complexity and heterogeneity of the tumor microenvironment frequently drive therapeutic resistance through mechanisms such as compensatory signaling pathway activation and local immunosuppressive adaptations. Second, monotherapy approaches carry risks of systemic toxicity due to off-target effects on homeostatic processes mediated by VEGF and CSF-1R in normal tissues, potentially compromising treatment safety. Furthermore, effective clinical implementation may require a synergistic combination with complementary targeted therapies or immunomodulatory agents, necessitating optimization of dosing schedules and therapeutic sequences to maximize efficacy while minimizing overlapping toxicities. Notably, the multilayered challenges of pathway crosstalk, unintended immunosuppression, tumor heterogeneity, and adaptive resistance mechanisms collectively hinder successful clinical translation. The next generation of CAC therapies will hinge on dismantling the tumor-promoting macrophage niche through mechanism-guided combinations, underpinned by robust biomarker platforms and smart delivery technologies. By addressing resistance drivers while preserving immune competence, such strategies may transform the CAC treatment paradigm from broad suppression to microenvironment-specific reprogramming. Collaborative efforts across immunology, bioengineering, and computational biology will be critical to navigate this complexity and deliver clinically impactful solutions.
